# Acute compartment syndrome of the thigh after total knee arthroplasty: a case report

**DOI:** 10.1186/s13256-024-04378-6

**Published:** 2024-02-20

**Authors:** Marie Schuster, Tobias Kappenschneider, Matthias Meyer, Dominik Emanuel Holzapfel

**Affiliations:** Department of Orthopaedic Surgery, University of Regensburg, Asklepios Klinikum, Bad Abbach, Germany

**Keywords:** Acute compartment syndrome, Total knee arthroplasty, Complications, Case report

## Abstract

**Introduction:**

Acute compartment syndrome of the thigh after total knee arthroplasty is a rarely described complication. After the assessment of the diagnosis, immediate surgical intervention is necessary to prevent further tissue damage. Since only a few cases have been described and because of the high complication rate, early detection is essential for ensuring patient outcomes.

**Case presentation:**

After total knee arthroplasty in a high-volume university hospital, a 57-year-old Caucasian female patient experienced strong, disproportional pain in the ventromedial thigh of the affected leg, which did not respond to an adequate adjustment in pain medication. Imaging revealed a distinct swelling of the vastus intermedius muscle. This resulted in acute compartment syndrome of the thigh, which was immediately surgically treated. Apart from receiving surgery distal from the affected compartment and continuous intake of acetylsalicylic acid, the patient had no risk factors for developing compartment syndrome. The patient’s recovery was uneventful, with timely wound closure and discharge to outpatient care without significant functional limitations.

**Conclusion:**

Acute compartment syndrome of the thigh represents a rare, but severe complication that can occur after orthopedic surgery. In our case, no triggering factors for the development of acute compartment syndrome, such as the use of a tourniquet, were detected. Even in unusual locations, compartment syndrome should be considered as a differential diagnosis. With sufficient evidence, immediate fasciotomy should be indicated.

## Introduction

Two days after undergoing total knee arthroplasty at a high-volume university hospital, a patient experienced strong, disproportional pain in the ventromedial thigh of the affected leg, which did not respond to an adequate adjustment in pain medication. Imaging revealed a distinct swelling of the vastus intermedius muscle. This resulted in acute compartment syndrome of the thigh, which was immediately treated surgically. Apart from receiving surgery distal to the affected compartment and a continuous intake of acetylsalicylic acid, the patient had no risk factors for the development of a compartment syndrome. The subsequent course was free of complications and wound closure was performed in a timely manner. The patient was discharged for follow-up treatment without any significant functional limitations.

Acute compartment syndrome of the thigh is a rare, but severe complication that can occur after interventions on the extremities. Even in uncommon locations, it is important to consider compartment syndrome as a differential diagnosis. If there is sufficient evidence, immediate fasciotomy should be performed.

## Case presentation

A 57-year-old Caucasian female nurse presented for elective total knee arthroplasty (TKA) due to osteoarthritis in our high-volume university hospital. Previous conservative treatment with physiotherapy and antiinflammatory medication had failed. She had a medical history of obesity [body mass index (BMI) of 33.4], type II diabetes, hypertension, and had previously undergone percutaneous coronary intervention because of coronary heart disease. Prior to the surgery, the range of motion (ROM) of her right knee was measured at 0-10-90° of extension/flexion and showed a varus leg axis. The patient reported load-related pain.

**Fig. 1:**
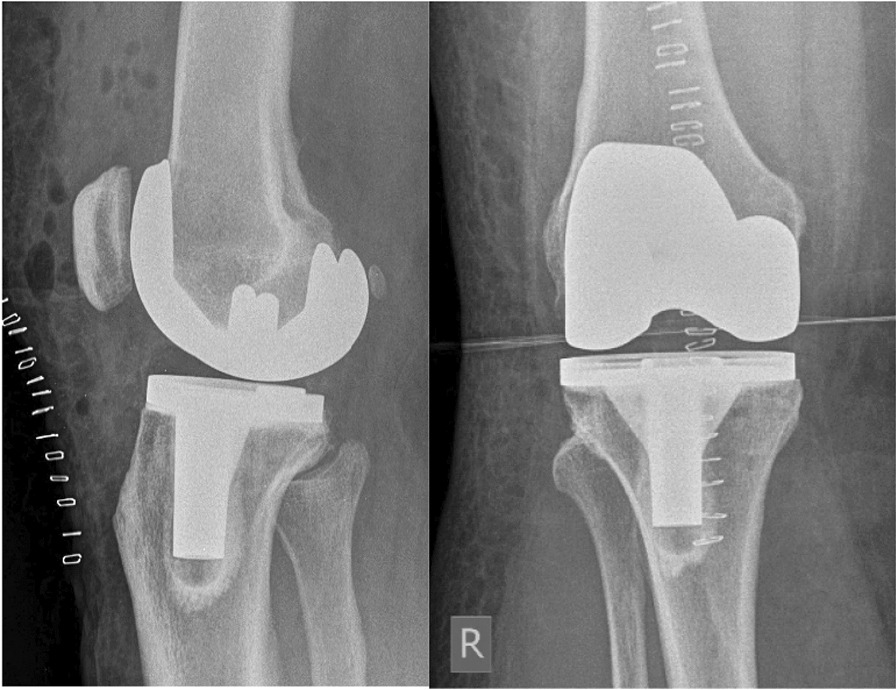
Postoperative X-ray control following the implantation of right total knee arthroplasty

The elective surgery was performed under spinal anesthesia (Fig. 1). During the navigation-based procedure, jigs were placed according to the standard operating procedure distal femoral medioventral and tibial proximal ventral. For local analgesia and hemostasis, 200 mg of ropivacaine 0.2% with 1 mg of adrenaline was applied dorsal of the medial and lateral condyle, in the area of the notch, collateral ligaments, synovialis, ventral capsule, and Hoffa’s fat pad. An additional 100 mg of 0.2% ropivacaine was applied subcutaneously. Furthermore, 3 g of tranexamic acid was applied intraarticularly. Tourniquet control was not used, nor was a drain inserted. The surgery was free of complications. On the same day, the patient underwent mobilization and began physiotherapy in accordance with our in-house FAST-Track protocol.

**Fig. 2:**
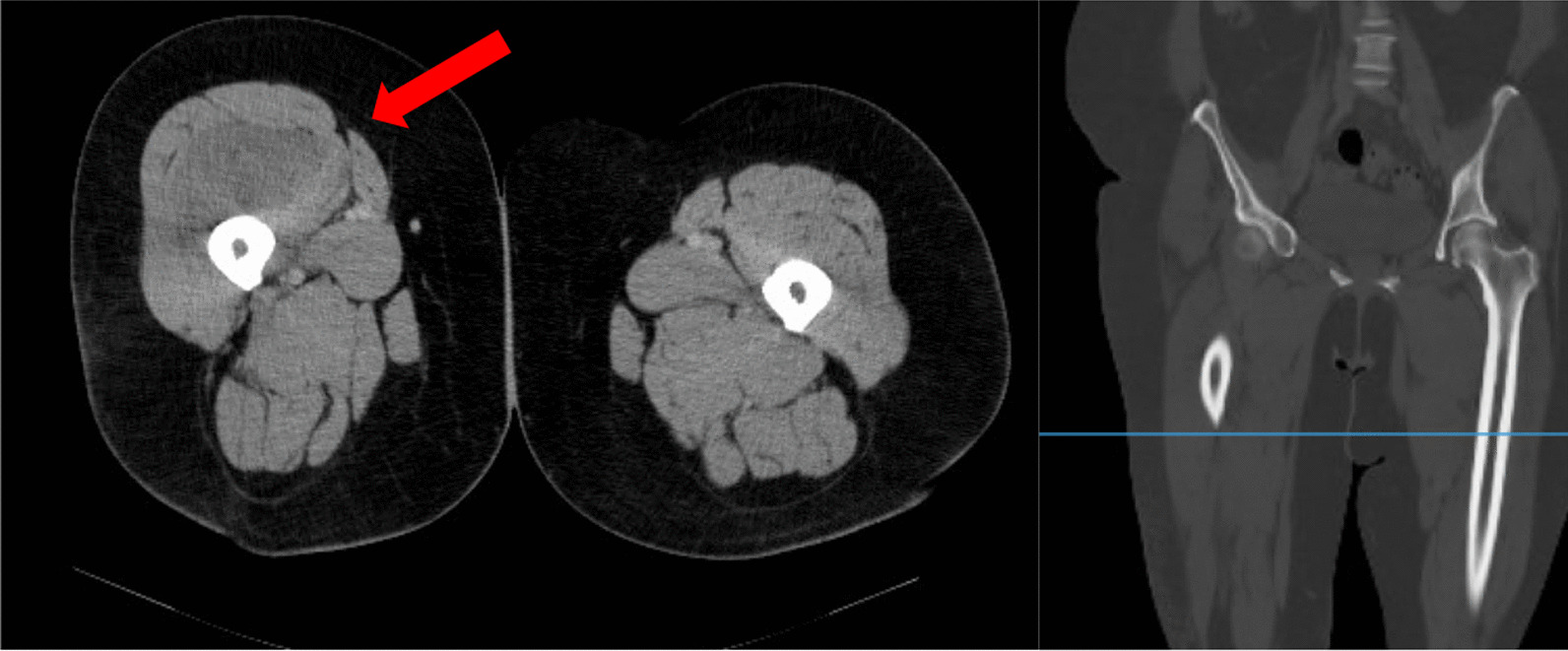
CT pelvic and leg imaging with contrast medium from postoperative day 2 (red arrow: clear signal alteration and circumferential increase in the vastus intermedius muscle in a side-by-side comparison)

The day after the surgery, the patient reported severe pain in her right leg, which necessitated an adjustment and an increase in her pain medication. After individual adjustment of her oral pain medication, the standard physiotherapy protocol was continued. On the second day after surgery, the patient experienced massive pain in her right middle thigh that was unresponsive to additional opioid use. The pain worsened with flexion of her leg. She showed a circumferential increase and pain on palpation. Because of the atypical location and the difficult physical examination due to obesity, an ultrasound was performed to rule out thrombosis. However, the examination was complicated by the patient’s obesity and thus ambiguous. After interdisciplinary consultation with our internal and radiological specialists, we initiated a computed tomography (CT) scan of the right thigh. A CT scan provides insight into the affected site, revealing complications such as thrombosis, hematoma, or fractures. Due to the rapid availability, we performed a CT scan and discussed the results immediately afterwards. The CT scan revealed signal alteration and massive swelling of the musculus vastus intermedius without signs of hematoma or acute bleeding (Fig. 2). Based on the physical examination and radiological evidence of an increase in volume in the right thigh, acute compartment syndrome was suspected, and immediate surgical intervention was indicated. Compartment pressure measurement was not performed as it would have delayed surgery. Immediately, a dermatofasciotomy of the right ventral thigh was performed.

We opened the fascia using a lateral access, followed by extensive debridement of all layers and enclosure via vacuum-assisted closure device (VAC). Significant swelling of the musculus vastus intermedius was observed during the procedure. There were no signs of hematoma or active bleeding, and no connection was found from the affected areas to the initial surgical site. Histological inspection revealed necrotic areas in the resected tissue.

**Fig. 3:**
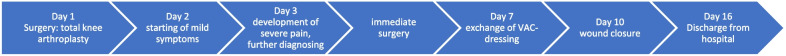
Timeline

After the surgery, the patient was immediately free of pain. We performed an exchange of the VAC-dressing 6 days after the initial surgery and successfully closed the wound on day 9. The further course was without complications, and our patient did not experience any functional deficiencies due to muscle debridement. She was discharged from our hospital to follow-up treatment with a ROM of extension/flexion of 0-0-60°. A total of 6 weeks after surgery, she could flex her knee to 100° and reported no pain or other issues (Fig. 3).

## Discussion

Acute compartment syndrome is a pathology that is mostly observed after fractures or complex trauma of the soft tissue of the distal extremity, predominantly the lower leg [1, 2]. It is classified by an acute increase in the pressure inside a muscle compartment, resulting in hypoxia of the affected tissue due to decreased perfusion pressure [1]. Symptoms include excessive therapy-resistant pain, decreased sensibility and motor function, and, in advanced stages, pulselessness [3].

The diagnosis is based on the patient’s medical history and clinical examination. Further technical examinations may be necessary for clarification. Risk factors should be evaluated in the medical history. During clinical examination, typical symptoms such as disproportional pain, pain unresponsive to medication, swelling, pain on passive stretch, pallor, paresthesia, and paresis should be observed [4]. Pulselessness can also be considered a differential diagnostic sign for acute arterial occlusion [5]. Whereas our patient experienced pain that was unresponsive to pain medication, circumferential increase and pain on palpation or passive stretch, we wanted to rule out thrombosis as a differential diagnosis. Swelling and pain in the quadriceps muscle after TKA are common postsurgical symptoms. However, the patient's symptoms persisted despite interventions according to our standard protocol, prompting further diagnostic investigation. Due to the patient’s obesity, the ultrasound performed was inconclusive, so we held an interdisciplinary round. A CT-scan provides insight into the affected site, revealing complications such as thrombosis, hematoma, or fractures. Due to the rapid availability, we performed a CT scan and discussed the results immediately afterwards. The images clearly indicated the need for surgery, and the patient was prepared accordingly.

Compartment pressure measurement can be used to diagnose compartment syndrome. In the case of compartment syndrome of the lower leg, limit values for total pressure, relative pressure difference to the diastolic blood pressure, and changes in pressure are described [1]. Pressure measurements have a negative predictive value of 99%, making them a reliable resource for decision-making [6]. However, it is important to note that limit values have only been investigated and validated for the lower leg; there is inadequate data on pressure or pressure changes in the thigh [3]. Conducting a measurement would have prolonged the time until surgery and would have only been of academic interest. Furthermore, many systems cannot be used on thighs due to the limited availability of needle sizes.

Therapy usually involves dermatofasciotomy surgery to relieve affected and threatened compartments [7]. Early intervention is crucial for preserving the affected tissue. The severity of clinical findings correlates with the risk of developing irreversible and extensive tissue damage [8]. Furthermore, progressive tissue damage heightens the risk of subsequent complications, such as rhabdomyolysis leading to acute kidney failure or electrolyte imbalances [9]. During the operation, we were unable to identify the cause of the muscle swelling or hematoma. The histological examination of the resected muscle tissue revealed necrotic areas, but no additional complications occurred.

Risk factors for compartment syndrome include previous adequate trauma, age under 35 years, male sex, crush and extensive soft tissue trauma, oral anticoagulation medication, long surgery duration, use of tourniquets, peripheral arterial occlusive disease, and obesity [10, 11]. In our case, no triggering factor for the development of a compartment syndrome was identified. For instance, we did not use tourniquet during the procedure and the surgery duration was kept short. Treatment with antiplatelet agents and obesity are considered to be risk factors. Finally, swelling of the muscle caused an increase of intercompartmental volume, but the origin of this change has not been conclusively determined.

**Fig. 4:**
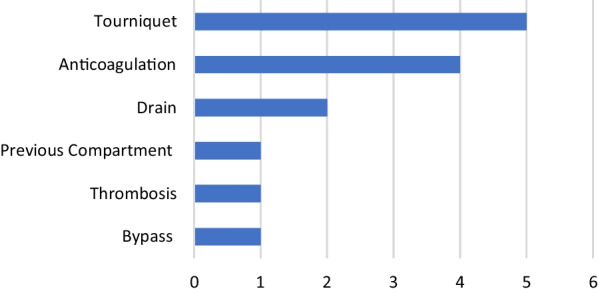
Triggering factors reported in the literature: use of a tourniquet, anticoagulation, use of drains, previous compartment syndrome, postoperative thrombosis *alio loco*, and bypass within the surgical area

The compartment syndrome of the thigh is rarely found in the literature and represents a rare complication after primary TKA. Our research uncovered 14 cases presenting a compartment syndrome of the thigh after endoprosthetic surgery of the lower limb. In each case, potential triggering factors for the development of compartment syndrome were found [12–17]. However, none of these triggering factors were applicable in our specific scenario (Fig. 4).

Thanks to interdisciplinary cooperation, the diagnosis was established quickly, and treatment started early. The good crosslinking between orthopedics, radiology, and anesthesia allowed for the prompt diagnosis and treatment of this uncommon complication.

Early therapy could prevent long-term sequelae. The postinterventional outcome of our patient did not decrease in the long term.

## Conclusion

Atypical sites, such as the thigh, should not distract from the suspected diagnosis if clinical examination is presented typically with inadequate pain and an increase in pain with passive movement. Early diagnosis and surgical intervention by decompression of the affected and threatened compartments are crucial for ensuring the vitality of the muscle tissue. Pressure measurement can be a useful tool for aiding in decision-making when diagnosing, but it has not yet been established for the thigh.

## Data Availability

Data sharing is not applicable to this article as no datasets were generated or analysed during the current study.

## Abbreviations

BMI: Body mass index
CT: Computed tomographic
ROM: Range of motion
TKA: Total knee arthroplasty
VAC: Vacuum-assisted closure device
